# *Synechococcus* sp. Strain PCC7002 Uses Sulfide:Quinone Oxidoreductase To Detoxify Exogenous Sulfide and To Convert Endogenous Sulfide to Cellular Sulfane Sulfur

**DOI:** 10.1128/mBio.03420-19

**Published:** 2020-02-25

**Authors:** Daixi Liu, Jiajie Zhang, Chuanjuan Lü, Yongzhen Xia, Huaiwei Liu, Nianzhi Jiao, Luying Xun, Jihua Liu

**Affiliations:** a Institute of Marine Science and Technology, Shandong University, Qingdao, China; b State Key Laboratory of Microbial Technology, Shandong University, Qingdao, China; c Joint Lab for Ocean Research and Education at Dalhousie University, Shandong University and Xiamen University, Qingdao, China; d Institute of Marine Microbes and Ecospheres, Xiamen University, Xiamen, China; e School of Molecular Biosciences, Washington State University, Pullman, Washington, USA; Yonsei University

**Keywords:** sulfide:quinone oxidoreductase, persulfide dioxygenase, cyanobacteria, H_2_S detoxification, sulfane sulfur

## Abstract

Eutrophication and deoxygenation possibly occur in coastal waters due to excessive nutrients from agricultural and aquacultural activities, leading to sulfide accumulation. Cyanobacteria, as photosynthetic prokaryotes, play significant roles in carbon fixation in the ocean. Although some cyanobacteria can use sulfide as the electron donor for photosynthesis under anaerobic conditions, little is known on how they interact with sulfide under aerobic conditions. In this study, we report that *Synechococcus* sp. strain PCC7002 (PCC7002), harboring an *sqr* gene encoding sulfide:quinone oxidoreductase (SQR), oxidized self-produced sulfide to S^0^, present as persulfide and polysulfide in the cell. The Δ*sqr* mutant contained less cellular S^0^ and had increased expression of key genes involved in photosynthesis, but it was less competitive than the wild type in cocultures. Further, PCC7002 with SQR and persulfide dioxygenase (PDO) oxidized exogenous sulfide to tolerate high sulfide levels. Thus, SQR offers some benefits to cyanobacteria even under aerobic conditions, explaining the common presence of SQR in cyanobacteria.

## Abstract (precis)

Cyanobacteria are a major force for primary production via oxygenic photosynthesis in the ocean. A marine cyanobacterium, PCC7002, is actively involved in sulfide metabolism. It uses SQR to detoxify exogenous sulfide, enabling it to survive better than its Δ*sqr* mutant in sulfide-rich environments. PCC7002 also uses SQR to oxidize endogenously generated sulfide to S^0^, which is required for the proper expression of key genes involved in photosynthesis. Thus, SQR has at least two physiological functions in PCC7002. The observation provides a new perspective for the interplays of C and S cycles.

## INTRODUCTION

Oxygen contents in the ocean have been declining during the past decades (1, 2), and the oxygen minimum zones (OMZs) where oxygen level is low or zero, are spreading (3, 4). With ocean warming, decreased solubility of O_2_ and increased biological activities, including photosynthesis and respiration, lead to the expansion of OMZs (5). Eutrophication induced by excessive nutrient input coming from agriculture and aquaculture is a common phenomenon in coastal waters (6), contributing to the expansion of OMZs. Nutrient enrichment via natural processes such as coastal upwelling may also be associated with deoxygenation (7). Hypoxia has serious impacts on marine ecosystems (8, 9). The sporadic accumulation of sulfide (H_2_S, HS^−^, and S^2−^) in OMZs is also a noticeable aspect of hypoxia hazards (10, 11).

The toxic effect of sulfide is well known, inhibiting respiration by acting on cytochrome *c* oxidase in heterotrophic bacteria (12–15) and photosynthesis by binding to metalloproteins of photosynthesis system II (PSII) (16–19). Sulfide can reach high concentrations in specific habitats, such as hydrothermal vents and seeps (10) and coastal mudflats (11). Sulfide is mainly derived from sulfate as the terminal electron acceptor for organic mineralization by sulfur-reducing bacteria in OMZs (10).

Sulfide is easily oxidized under both aerobic and anaerobic conditions. Chemolithotrophic bacteria oxidize it under aerobic conditions, while sulfur-dependent photosynthetic bacteria oxidize it under anaerobic conditions, using the generated electrons for anaerobic photosynthesis (20–22). Recently, mammalian mitochondria and heterotrophic bacteria were reported to oxidize sulfide via a pathway involving two key enzymes, sulfide:quinone oxidoreductase (SQR) and persulfide dioxygenase (PDO) (23, 24). SQR oxidizes sulfide to polysulfide, which spontaneously reacts with glutathione (GSH) to produce glutathione persulfide (GSSH); PDO oxidizes GSSH to sulfite, which spontaneously reacts with polysulfide to produce thiosulfate (25). This pathway is common in heterotrophic bacteria (21).

SQRs are widely distributed in microorganisms as well as in animal mitochondria (21, 26, 27). SQR oxidizes sulfide to polysulfide and transfers electrons into the electron transport chain in mitochondria (28), heterotrophic bacteria (21), chemolithotrophic bacteria (29, 30), and photolithotrophic bacteria (27, 31, 32). SQRs are grouped into six types based on sequence and structural analyses (33, 34). Besides being further oxidized, polysulfide (H_2_Sn) can be converted into other forms of cellular sulfane sulfur, including protein presulfidation at Cys residues in bacteria (35). Sulfane sulfur is zero valence sulfur in various forms, such as persulfide (RSSH), polysulfide (RSSnH and RSSnR, *n* ≥ 2), and elemental sulfur. Sulfane sulfur can act as either an electrophile or a nucleophile (36). The nucleophilic property allows cells to resist reactive oxygen species, and the electrophilic property causes protein persulfidation, affecting enzyme activities or signaling (37, 38).

The cyanobacteria play a vital role in global primary production as the most ancient and abundant phytoplankton in the ocean (39, 40). Cyanobacteria possess both photosynthesis system I (PSI) and photosynthesis system II (PSII), and they usually perform oxygenic photosynthesis but can do anoxygenic photosynthesis (41, 42). They are effective participants in marine carbon cycles (43). Cyanobacteria are found to inhabit multiple marine habitats, and *Synechococcus* spp. dominate the picocyanobacterial communities in eutrophic coastal and mesotrophic open ocean waters (44, 45). In some habitats, they will encounter sulfide. The sulfur cycle is generally coupled with the carbon cycle (46–48); however, it is still unclear how sulfide affects *Synechococcus* spp. and how they deal with it.

Here, we report that *Synechococcus* sp. strain PCC7002 (PCC7002) used SQR to oxidize self-produced sulfide to polysulfide, maintaining a relatively high level of cellular sulfane sulfur, which offers growth advantages to the wild type over the Δ*sqr* mutant. Further, PCC7002 also survived better in sulfide-rich environments via the detoxification role of SQR and PDO. The two enzymes collectively oxidize sulfide to sulfite and thiosulfate, resembling the newly reported pathway in heterotrophic bacteria (25).

## RESULTS

### PCC7002 uses SQR to oxidize endogenous sulfide.

**FIG 1:**
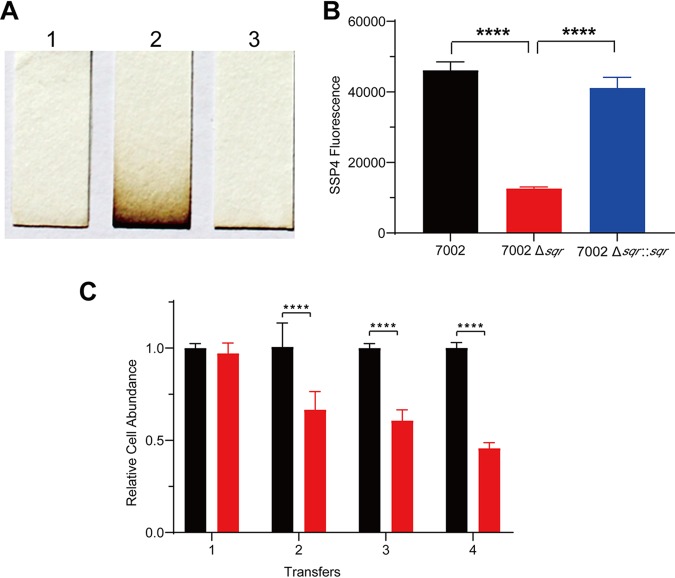
The effect of SQR on H_2_S accumulation, cellular sulfane sulfur, and competitiveness of PCC7002 with its mutant. (A) H_2_S accumulation during culture as detected in the gas phase with lead-acetate paper. 1, PCC7002; 2, PCC7002Δ*sqr*; 3, PCC7002Δ*sqr*::*sqr*. (B) Sulfane sulfur contents of PCC7002 (black bar), PCC7002Δ*sqr* (red bar), and PCC7002Δ*sqr*::*sqr* (blue bar) were detected by using SSP4 (sulfane sulfur probe 4). (C) Competition experiment of PCC7002 and PCC7002Δ*sqr*. The abundance of PCC7002 (black bars) showed an apparent advantage over PCC7002Δ*sqr* (red bars) after transfer 4 times. The mixing of two strains at equal cell numbers was defined as the first transfer. The time interval between each transfer was 48 h, and real-time quantitative PCR (qPCR) was done at 48 h. All data are averages from three samples with standard deviation (error bar). The experiment was repeated at least three times. ****, *P* < 0.0001 (paired *t* test).

PCC7002 contains one *sqr* gene (CyanoBase: SYNPCC7002_G0075), encoding a type I SQR. To generate the Δ*sqr* mutant (see Table S1 in the supplemental material), the gene was replaced with a kanamycin resistance gene via homologous recombination (Fig. S1). The mutant was complemented with the *sqr* gene inserted on the chromosome of the mutant at a neutral site (CyanoBase: SYNPCC7002_A0933) (Table S1). The wild type and the complementation strain PCC7002 Δ*sqr*::*sqr* did not accumulate sulfide, but the mutant did (Fig. 1A). We also checked cellular sulfane sulfur contents by using sulfane sulfur probe 4 (SSP4), which releases a fluorescent compound after reacting with sulfane sulfur (49). The Δ*sqr* mutant contained a sharply decreased level of cellular sulfane sulfur in comparison with the wild type and PCC7002 Δ*sqr*::*sqr* (Fig. 1B). The results indicate that PCC7002 uses SQR to oxidize endogenously produced sulfide, allowing the bacterium to keep a relatively high level of cellular sulfane sulfur.

10.1128/mBio.03420-19.1 FIG S1 Genetic analysis of *sqr* in PCC7002. The inactivation of *sqr* was verified by PCR. 1, PCC7002; 2, PCC7002Δ*sqr*. Download FIG S1, TIF file, 1.3 MB. Copyright © 2020 Liu et al. 2020 Liu et al. This content is distributed under the terms of the Creative Commons Attribution 4.0 International license.

10.1128/mBio.03420-19.7 TABLE S1 Strains and plasmids used in this study. Download Table S1, DOCX file, 0.03 MB. Copyright © 2020 Liu et al. 2020 Liu et al. This content is distributed under the terms of the Creative Commons Attribution 4.0 International license.

### PCC7002 has a competitive advantage over the Δ*sqr* mutant in coculture.

To investigate the effect of SQR, we monitored the growth and photosynthesis of the wild type and the Δ*sqr* mutant. The deletion of *sqr* had no effect on the growth of PCC7002 (Fig. S2). We then cocultured PCC7002 and PCC7002Δ*sqr* to access their competitive capacity. The relative abundance of PCC7002 and PCC7002Δ*sqr* in the coculturing system was detected by real-time quantitative reverse transcription-PCR (qRT-PCR) with specific primer pairs (Table S2). Different ratios of PCC7002 and PCC7002Δ*sqr* were mixed, and the qRT-PCR results correlated with the ratios accordingly (Fig. S3), confirming the approach. PCC7002 and PCC7002Δ*sqr* showed little difference in the coculture system during the initial 48 h; however, the advantage of the wild type became apparent after continuous transfer (Fig. 1C). We also cultured PCC7002 and PCC7002Δ*sqr* separately using the same method, but results showed no difference between the wild type and the mutant (Fig. S4).

10.1128/mBio.03420-19.2 FIG S2 Growth curves of PCC7002 and PCC7002Δ*sqr*. There is no apparent difference between PCC7002 (black circles) and PCC7002Δ*sqr* (red squares) in A+ medium. Download FIG S2, TIF file, 0.1 MB. Copyright © 2020 Liu et al. 2020 Liu et al. This content is distributed under the terms of the Creative Commons Attribution 4.0 International license.

10.1128/mBio.03420-19.3 FIG S3 Relative abundance of PCC7002 and PCC7002Δ*sqr* in mixed culture. PCC7002 (black bars) and PCC7002Δ*sqr* (red bars) are mixed in different proportions and detected by using qRT-PCR. The ratios of PCC7002 and PCC7002Δ*sqr* in the mixture are 4:1 (A), 2:1 (B), 1:1 (C), 1:2 (D), 1:4 (E), and 1:0 (F). Download FIG S3, TIF file, 0.4 MB. Copyright © 2020 Liu et al. 2020 Liu et al. This content is distributed under the terms of the Creative Commons Attribution 4.0 International license.

10.1128/mBio.03420-19.4 FIG S4 Relative abundance of PCC7002 and PCC7002Δ*sqr* cultured separately. PCC7002 (black bars) and PCC7002Δ*sqr* (red bars) showed little difference when cultured separately in A+ medium. Download FIG S4, TIF file, 0.3 MB. Copyright © 2020 Liu et al. 2020 Liu et al. This content is distributed under the terms of the Creative Commons Attribution 4.0 International license.

10.1128/mBio.03420-19.8 TABLE S2 Primers used in this study. Download Table S2, DOCX file, 0.03 MB. Copyright © 2020 Liu et al. 2020 Liu et al. This content is distributed under the terms of the Creative Commons Attribution 4.0 International license.

### The effects of *sqr* inactivation on the photosynthesis process.

**FIG 2:**
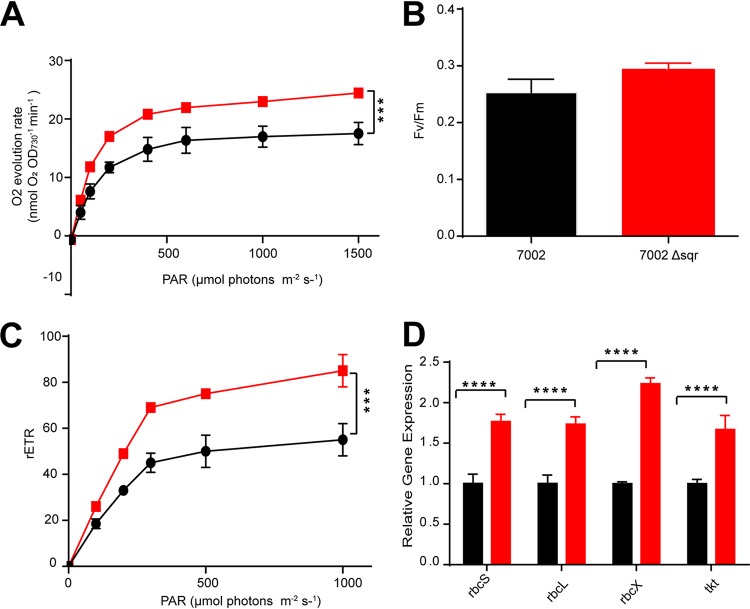
The effects of *sqr* inactivation on photosynthesis and related gene expression. (A) The Δ*sqr* mutant (red squares) showed a higher oxygen evolution rate than the wild type (black circles). PAR, photosynthetically active radiation. ***, *P* < 0.001 (two-way analysis of variance [ANOVA]). (B) The Fv/Fm value of the Δ*sqr* mutant (red bar) showed a 16% increase over that of the wild type (black bar). (C) The relative electron transport rate (rETR) of the Δ*sqr* mutant (red squares) increased over that of the wild type (black circles). ***, *P* < 0.001 (two-way ANOVA). (D) The *rbcS*, *rbcL*, *rbcX*, and *tkt* genes in PCC7002Δ*sqr* (red bars) were all upregulated compared with the wild type (black bars) as detected by qRT-PCR. All data are averages from six samples with standard deviation (error bar). The experiment was repeated at least three times. ****, *P* < 0.0001 (paired *t* test).

The photosynthesis parameters of the wild type and the Δ*sqr* mutant were tested. The Δ*sqr* mutant had a higher oxygen evolution rate than that of the wild type (Fig. 2A). The increased oxygen evolution rate upon the Δ*sqr* mutant was consistent with an acceleration of H_2_O oxidation to make up for the loss of H_2_S oxidation. The maximal PSII quantum yield was calculated as Fv/Fm, a measure of the conversion efficiency of the light energy of the PSII reaction center. Here, the Fv/Fm value showed little difference between the wild type and the mutant (Fig. 2B). The relative electron transport rate (rETR) increased in the mutant (Fig. 2C). Thus, photosynthesis efficiency was increased in the Δ*sqr* mutant. However, the apparently increased efficiency in photosynthesis did not affect the growth (Fig. S2). We then checked the expression levels of major genes involved in photosynthesis in the wild type and the Δ*sqr* mutant. PCC7002 had three *psbA* genes, encoding the isoforms of D1 protein, a key protein of the PSII core, and the isoforms allowed cyanobacteria to adapt to different light intensities. The expression levels of *psbA1*, *psbA2*, and *psbA3* were all upregulated (1.5- to 2-fold) in the Δ*sqr* mutant (Fig. S5). The *rbcX*, *rbcL*, and *rbcS* genes encoding RuBisCO and the *tkt* gene encoding transketolase of the Calvin-Benson-Bassham (CBB) cycle all showed apparent transcriptional increases (>2-fold) in the Δ*sqr* mutant in comparison with the wild type (Fig. 2D). The mRNA level of *petA* encoding apocytochrome *f* precursor increased by 1.8-fold, and the transcripts of *petC* encoding a Rieske-FeS protein increased by 1.5-fold in the Δ*sqr* mutant. However, the transcripts of the *petB* gene encoding cytochrome *b*_6_ showed little change (Fig. S5). Overall, the qRT-PCR results indicated that the inactivation of *sqr* in PCC7002 affected many physiological processes, including PSII, the CBB cycle, and the photosynthetic electron transport chain (Fig. 2D and Fig. S5).

10.1128/mBio.03420-19.5 FIG S5 Relative expression of photosynthesis-related genes. (A) The *psbA1*, *psbA2*, and *psbA3* genes were approximately 1.5- to 2-fold higher in the *sqr* mutant than in the wild type. (B) The *petA* and *petB* genes were upregulated, but the *petC* gene was unchanged in the mutant. Download FIG S5, TIF file, 1.0 MB. Copyright © 2020 Liu et al. 2020 Liu et al. This content is distributed under the terms of the Creative Commons Attribution 4.0 International license.

### SQR plays a detoxification role in PCC7002.

**FIG 3:**
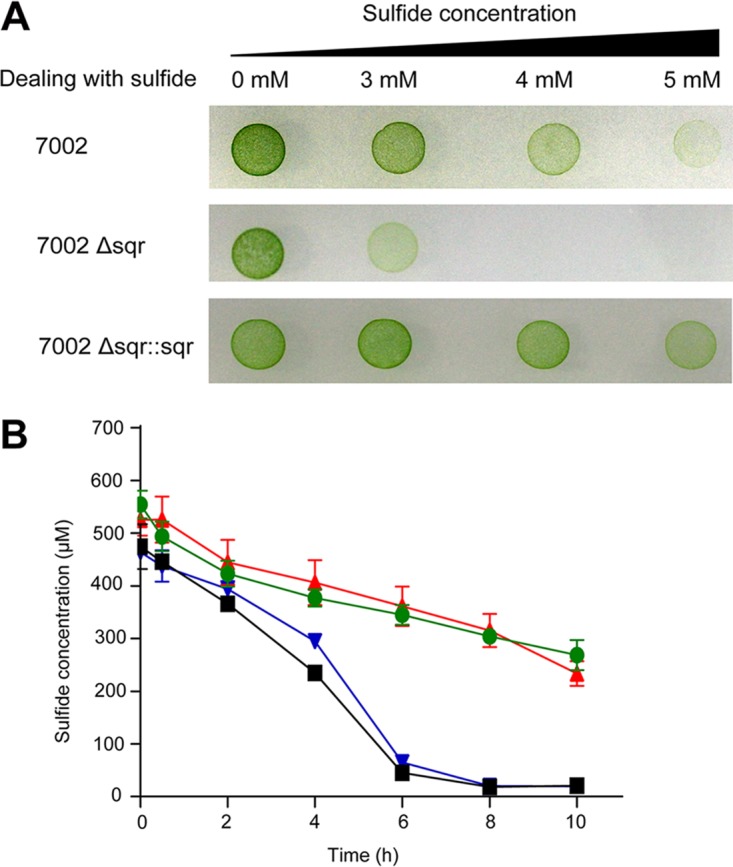
SQR allows PCC7002 to survive sulfide exposure by oxidizing it. (A) The effect of sulfide on the growth of PCC7002 and its mutants. All three strains survived exposure to 3 mM sulfide for 6 h. Only the wild type and the complementation strain survived exposure to 4 mM or 5 mM sulfide for 6 h. (B) Sulfide oxidation by PCC7002 and its mutants. The cell suspensions (OD_730_ of 8 in 50 mM Tris buffer, pH 8.0) of PCC7002 (black squares) and PCC7002Δ*sqr*::*sqr* (blue inverted triangles) oxidized the added NaHS within 8 h, while sulfide in the Δ*sqr* mutant (red triangles) suspension decreased in a similar way as that in 50 mM Tris buffer (green circles). All data are averages from at least three samples with standard deviation (error bar). The experiment was repeated at least three times.

We treated the wild type, the Δ*sqr* mutant, and the complementation strain with different concentrations of NaHS for 6 h and then washed, diluted, and inoculated cells onto the A+ medium for incubation under 50 μmol photons m^−2^ s^−1^ at 30°C. Without sulfide, all the three strains grew well on the A+ agar plate. After treatment with 3 mM NaHS, the Δ*sqr* mutant grew worse than the wild type and the complementation strain (Fig. 3A). The growth of the Δ*sqr* mutant was fully inhibited while the wild type and the complementation strain were partly inhibited after treatment with 4 mM and 5 mM NaHS.

Whether PCC7002 oxidized exogenous sulfide was also tested. Cells at log phase of growth (optical density at 730 nm [OD_730_] = 1) were harvested and resuspended in 50 mM Tris buffer (pH 8.0) at an OD_730_ of 8. Without induction, all the three strains (PCC7002, PCC7002Δ*sqr*, and PCC7002Δ*sqr*::*sqr*) showed no oxidation activity (data not shown). After induction with 2 mM NaHS for 6 h, cell suspensions of the wild type and the complementation strain showed apparent activity with about 500 μM NaHS being fully oxidized in 8 h (Fig. 3B). However, the Δ*sqr* mutant showed a similar oxidation rate as the buffer control (Fig. 3B). Thus, SQR plays a detoxification role in PCC7002 by oxidizing excessive sulfide.

### Recombinant Escherichia coli with SQR and PDO from PCC7002 oxidized sulfide to sulfite and thiosulfate.

**FIG 4:**
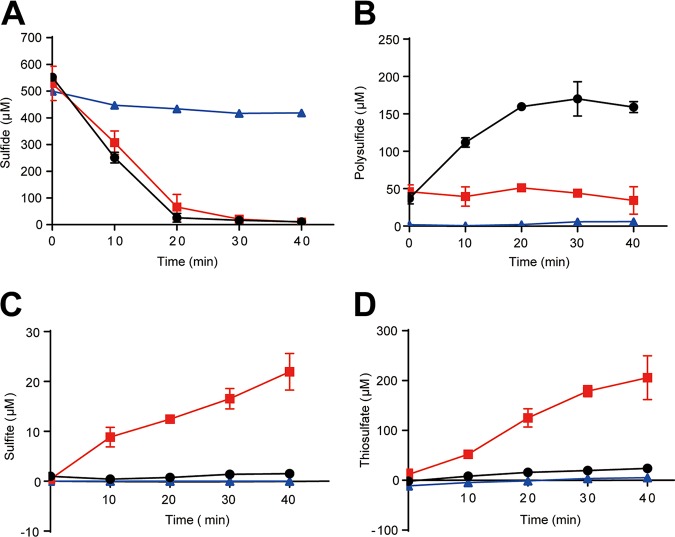
Sulfide oxidation by recombinant E. coli. Sulfide (A), polysulfide (B), sulfite (C), and thiosulfate (D) were consumed or produced by E. coli(pBBR5-*sqr*) (black circles), E. coli(pBBR5-*sqr-pdo*) (red squares), and E. coli(pBBR1MCS5) (blue triangles, control). All data are averages from at least three samples with standard deviation (error bar). The experiment was repeated at least three times.

PCC7002 also has a persulfide dioxygenase (PDO). We constructed recombinant strains E. coli(pBBR5-*sqr*), containing only *sqr*, and E. coli(pBBR5-*sqr*-*pdo*), containing both *sqr* and *pdo*. The cell suspensions of E. coli(pBBR5-*sqr*) rapidly oxidized 500 μM sulfide to about 250 μM polysulfide in 20 min with no apparent production of sulfite and thiosulfate (Fig. 4A to D). E. coli(pBBR5-*sqr*-*pdo*) oxidized 500 μM sulfide to about 22 μM sulfite and 205 μM thiosulfate with elevated levels of polysulfide (>50 μM) (Fig. 4A to D). The results were similar to recombinant E. coli with *sqr* and *pdo* from heterotrophic bacteria (25).

### The presence of *sqr* and *pdo* in cyanobacteria.

**FIG 5:**
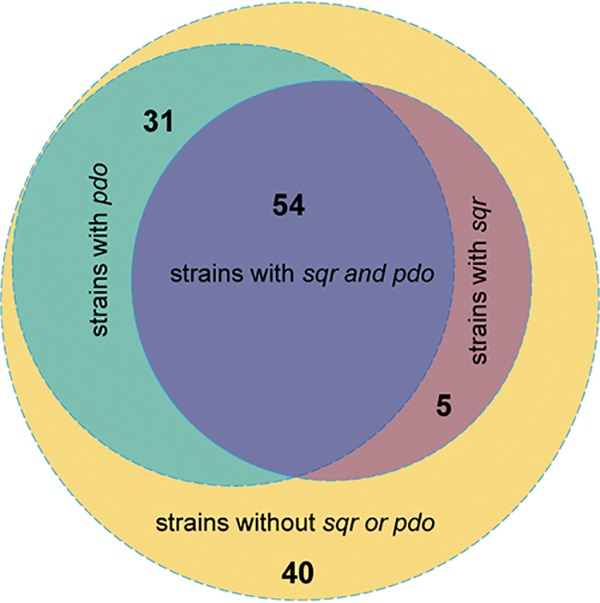
The distribution of SQRs and PDOs in sequenced cyanobacterial genomes. Fifty-nine of 130 sequenced cyanobacterial genomes possessed SQR, and 85 of 130 possessed PDO. Fifty-four cyanobacterial genomes possessed both SQR and PDO.

The previously reported SQRs and PDOs were used to search for potential SQRs and PDOs from 130 sequenced cyanobacterial genomes (NCBI database, updated to 17 June 2019) (21). Sixty-seven SQRs from 59 cyanobacterial genomes and 101 PDOs from 85 cyanobacterial genomes were identified after checking via phylogenetic analysis; 54 genomes possessed both SQRs and PDOs (Fig. 5). All the cyanobacterial SQRs belonged to the type I and type II SQRs (Fig. S6).

10.1128/mBio.03420-19.6 FIG S6 Phylogenetic analysis of cyanobacterial SQRs. All the cyanobacterial SQRs belong to type I or type II. Reference proteins are listed below, with the organism origin and accession number: FCSD1, Allochromatium vinosum (WP_012970307.1); FCSD2, *Beggiatoa* sp. strain PS (ABBZ01000762.1); sqrI-1, Glaciecola nitratireducens (WP_014108326.1); sqrI-2, Thiobacillus denitrificans (AAM52227.1); sqrI-3, *Geitlerinema* sp. strain PCC 9228 (WP_071516517.1); sqrII-1, Meiothermus ruber (WP_013015093.1); sqrII-2, Pseudomonas aeruginosa (WP_023131969.1); sqrIII-1, Archaeoglobus fulgidus (WP_010878064.1); sqrIII-2, Chlorobaculum tepidum (WP_010932556.1); sqrIV-1, Lutibacter profundi (WP_068209874.1); sqrVI_1, Halothiobacillus neapolitanus (WP_012823197.1). Download FIG S6, TIF file, 1.5 MB. Copyright © 2020 Liu et al. 2020 Liu et al. This content is distributed under the terms of the Creative Commons Attribution 4.0 International license.

## DISCUSSION

**FIG 6:**
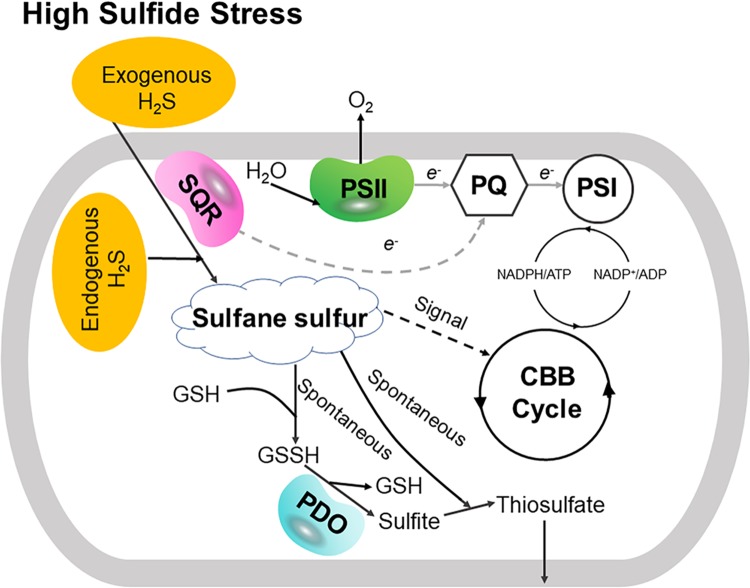
SQR helps PCC7002 deal with high sulfide and improves its growth fitness. On the one hand, SQR oxidizes exogenous H_2_S to sulfane sulfur, which spontaneously reacts with GSH and generates GSSH. Then, PDO oxidizes GSSH to sulfite and generates a new GSH. At last, sulfite spontaneously reacts with sulfane sulfur to generate thiosulfate, which is transported out of the cell. Hence, SQR coupling with PDO plays a detoxification role by oxidizing H_2_S to sulfite and thiosulfate. On the other hand, SQR oxidizes endogenous H_2_S to sulfane sulfur, which may act as a signal molecule and participates in the regulation of photosynthesis, especially in the CBB cycle (the proposed signaling function of sulfane sulfur is indicated with a dashed black line). Meanwhile, H_2_S oxidation may donate to the photosynthetic electron transport (proposed electron transfer from SQR to plastoquinone [PQ] is indicated with a dashed gray line). As a result, SQR enables PCC7002 to increase its growth fitness as shown when culturing the wild type and the mutant in a mixture.

Our data indicate two physiological roles of SQR in PCC7002 (Fig. 6). First, SQR oxidizes endogenous H_2_S to polysulfide, which makes PCC7002 more competitive in coculture of the wild type and the Δ*sqr* mutant (Fig. 1). It is likely that polysulfide is converted to other species of cellular sulfane sulfur, participating in the regulation of key genes of photosynthesis as a signaling molecular. H_2_S has been proposed as a signaling molecule in bacteria since 2006 (50). Recently, it was shown that the signaling role of H_2_S is via sulfane sulfur, which is sensed by gene regulators that activate the genes involved in H_2_S oxidation in bacteria (51–53). OxyR also senses high levels of cellular sulfane sulfur to produce enzymes for its removal (54). Our evidence suggests that sulfane sulfur is likely involved in regulating photosynthetic genes (Fig. 2D; see also Fig. S5 in the supplemental material).

Second, SQR plays a detoxification role. Sulfide may accumulate at high concentrations in OMZs (10). With SQR, PCC7002 survives better than the Δ*sqr* mutant in the presence of high concentrations of sulfide (Fig. 3A). The detoxification also requires PDO that prevents the excessive accumulation of polysulfide, and the sulfide oxidation pathway is the same as in heterotrophic bacteria (25). Bioinformatics analysis shows that about 45% of the sequenced cyanobacterial genomes contain SQR (Fig. 5), while only 21% of the sequenced bacterial genomes contain SQR (21), indicating the wide distribution and importance of SQR in cyanobacteria. Thus, SQR may facilitate PCC7002 and possibly other cyanobacteria to adapt to changing environments and increase their growth fitness.

SQR may have a third role in cyanobacteria, allowing them to use H_2_S as the electron donor for photosynthesis. In the sulfur photosynthetic bacteria, SQR oxidizes H_2_S and passes the electrons to drive anaerobic photosynthesis (52). Some cyanobacteria also perform anoxygenic photosynthesis in sulfide-rich environments (18, 31, 41), partly because sulfide is a better electron donor and partly because sulfide at high concentrations inhibits photosystem II (16). We observed that the Δ*sqr* mutant had a higher oxygen evolution rate than that of the wild type (Fig. 2A). The observation may be a result of increased expression of photosynthetic genes or the oxidation of endogenously produced sulfide by SQR. Further investigation is needed to clarify whether sulfide provides electrons for photosynthesis under aerobic conditions. The physiological roles of SQR reported here may guide the investigation of cyanobacteria in ecological niches with varied O_2_ and sulfide contents, as many sequenced cyanobacterial genomes contain SQR (Fig. 5).

The source of endogenous H_2_S in PCC7002 is likely from the metabolism of cysteine or homocysteine, similar to H_2_S production by heterotrophic bacteria during normal growth (21), or from assimilatory sulfate reduction (55). The endogenous H_2_S has been shown to act as a signal molecule at low concentrations (12). Cellular sulfane sulfur, including polysulfide and GSSH, is believed to be the actual mediator of H_2_S signaling (56–59); sulfane sulfur can also protect Escherichia coli cells from oxidative stress (35).

When SQR is inactivated in PCC7002, an interesting phenomenon is that the growth advantage of PCC7002 occurs only in the coculture process (Fig. 1C and Fig. S2). This may be caused by the intracellular polysulfide coming from endogenous H_2_S oxidation by SQR. The mutant contains less sulfane sulfur (Fig. 1B) but has increased oxygen evolution rates and rETRs with elevated expression of photosynthesis genes (Fig. 2). The gene expression variation between the mutant and the wild type may also be a result of the lowered sulfane sulfur in the mutant, as sulfane sulfur is a known signaling molecule participating in many physiological processes in eukaryotes (58). And from our results, we infer that carbon and sulfur metabolisms can be linked via sulfane sulfur just like carbon and nitrogen metabolisms in cyanobacteria (60, 61). As we know, sulfane sulfur can perform as a reductant to help relieve oxidative stress (35, 37). Here, increased photosynthesis efficiency with lower sulfane sulfur content in the Δ*sqr* mutant may cause oxidative stress (62), thus making it less competitive in coculture. However, it is unclear how polysulfide regulates key genes involved in photosynthesis processes, such as PSII, the CBB cycle, and photosynthesis electron transport.

In summary, SQR helps PCC7002 to convert endogenous H_2_S to polysulfide, which is used to maintain a relatively high level of cellular sulfane sulfur, and to detoxify exogenous H_2_S. The relatively high cellular sulfane sulfur offers the wild type a growth advantage over the Δ*sqr* mutant in a mixed culture. The detoxification role of SQR may enable PCC7002 to survive better in OMZs, thus helping relieve deoxygenation by generating more O_2_. Thus, sulfide oxidation plays a vital role in the adaption process of PCC7002 and possibly other cyanobacteria. We present a new perspective for understanding the coupling of carbon and sulfur cycles, which are key components of the marine biogeochemical cycle.

## MATERIALS AND METHODS

### Strains and culture conditions.

PCC7002 cultures were grown in conical flasks containing medium A, supplemented with 1 mg of NaNO_3_ ml^−1^ (designated medium A+) (63) under continuous illumination of 50 μmol photons m^−2^ s^−1^ at 30°C. Glycerol (10 mM) was added as a supplement in the A+ medium to serve as the carbon and energy source. For mutant strains, the concentrations of appropriate antibiotics required by the mutant strains were as following: 100 g/ml kanamycin and 20 g/ml chloramphenicol. E. coli was cultured in LB medium at 37°C. The strains and plasmids used in this paper are all listed in Table S1 in the supplemental material.

### Strain construction.

The Δ*sqr* mutant of PCC7002 was constructed by homologous recombination. Two gene segments immediately upstream and downstream of the *sqr* gene (CyanoBase: SYNPCC7002_G0075), about 1,000 bp long, were amplified by PCR from genomic DNA of the wild-type strain with the primer sets *sqr*-del-1/*sqr*-del-2 and *sqr*-del-5/*sqr*-del-6 (Table S2). A kanamycin resistance cartridge was excised from the plasmid pET30a with the primers *sqr*-del-3/*sqr*-del-4. All the above three fragments were joined via fusion PCR, with the kanamycin resistance cartridge in the middle. Then, the fusion fragment was cloned into pJET1.2 blunt vector (Thermo, Beijing, China). The *sqr* gene was inserted at neutral site 1 (CyanoBase: SYNPCC7002_A0933) of PCC7002 to form the complementation strain (64). Wild-type cells were transformed with these constructs, and transformants were selected using appropriate antibiotics. The primers used in this paper are all listed in Table S2.

The *sqr* gene and *pdo* gene (CyanoBase: SYNPCC7002_A2866) of PCC7002 were also cloned and expressed in E. coli BL21(DE3). The fragment of *sqr* was amplified from the PCC7002 genome using primers pbr-*sqr*-F and pbr-*sqr*-R (listed in Table S1) containing 20-bp extensions overlapping the adjacent fragment. The *sqr* fragment was cloned into the plasmid pBBR1mcs-5 (pBBR5) by using the TEDA assembly (65). The plasmid pBBR5-*sqr*-*pdo* containing both *sqr* and *pdo* was constructed using the same protocol with primers listed in Table S1. The two plasmids pBBR5-*sqr* and pBBR5-*sqr*-*pdo* were transformed to E. coli BL21(DE3).

### Sulfide accumulation detection.

The method used for the detection of sulfide accumulation was the same as described previously (65, 66). PCC7002 cultures were transferred into 3 ml of A+ medium in a 15-ml glass tube and incubated with shaking for 96 h with lead acetate [Pb(Ac)_2_] paper strips at the top of the tube, which would turn black in the presence of sulfide as the production of PbS black precipitates.

### Toxicity analysis of sulfide.

PCC7002 and its mutants at log phase at an OD_730_ of 0.6 to 0.7 were treated with 3 mM, 4 mM, or 5 mM NaHS for 6 h in the sealed tubes. After incubation, cells were washed and resuspended in fresh A+ medium. The cells were diluted with A+ medium at an OD_730_ of 0.05, and 10 μl was placed on the A+ agar plate. The differences between PCC7002 and its mutants appeared after cultivation at 30°C under continuous illumination of 50 μmol photons · m^−2^ · s^−1^ for 7 days.

### Sulfide oxidation and product analysis.

Sulfide oxidation by PCC7002 and its mutants was assayed. Cells were harvested after induction with 2 mM NaHS for 6 h at log phase. They were resuspended in 50 mM Tris buffer (pH 8.0) at an OD_730_ of 8 after being washed twice with the same buffer. The reaction was performed in 30-ml serum bottles sealed with a rubber stopper to minimize the loss of sulfide. NaHS (0.5 mM) was added to the bottle to initiate the reaction.

Sulfide oxidation by recombinant *E.coli* was similar, but cells were harvested and resuspended to an OD_600_ of 2. The concentrations of sulfide, polysulfide, sulfite, and thiosulfate were analyzed as described previously (25, 66). Briefly, sulfide was detected by a colorimetric method; polysulfide, sulfite, and thiosulfate were derivatized with monobromobimane and then measured by high-performance liquid chromatography (HPLC) with a fluorescence detector.

### Competition experiment.

PCC7002 and PCC7002Δ*sqr* at log phase with an OD_730_ of 0.6 to 0.7 were transferred to fresh A+ medium to a final concentration with an OD_730_ of 0.05 each as a mixture. The total genomic DNA of the mixed culture was extracted and used as the template for real-time quantitative PCR (qPCR) analysis after culturing for 48 h. The abundance of the *sqr* gene was used as the marker for the relative abundance of PCC7002, and the abundance of the *kan* gene was used for PCC7002Δ*sqr*, in which the *sqr* gene was replaced by the *kan* gene. Then, the mixed culture was transferred to fresh A+ medium at an OD_730_ of 0.05 for another 48 h and analysis. The mixing of two strains at equal cell numbers was defined as the first transfer. The mixture was transferred 4 times.

### Endogenous sulfane sulfur analysis.

Sulfane sulfur probe 4 (SSP4) was used to detect endogenous sulfane sulfur as previously described (35, 49). Cells were harvested, washed, and resuspended in 50 mM Tris buffer (pH 8.0). Cells were disrupted by ultrasonication, and debris was removed by centrifugation at 12,500 × *g* for 10 min. SSP4 (10 μM) was added to the supernatant and incubated in the dark at 30°C for 15 min. The fluorescence resulting from the SSP4 reaction with sulfane sulfur was detected with excitation of 482 nm and emission of 515 nm by using a Synergy H1 microplate reader. The ﬂuorescence values were expressed as the intensity per milligram of protein.

### Oxygen evolution and chlorophyll fluorescence assay.

Cells at log phase with an OD_730_ of 0.6 to 0.7 were collected for oxygen evolution measurement via a Clark oxygen electrode (Chlorolab 2+; Hansatech, Norfolk, United Kingdom). The samples were illuminated under continuous stirring (900 rpm) at 30°C with increasing light intensities (from 0 to 1,000 μmol photons m^−2^ s^−1^). The rate of oxygen evolution was recorded continuously for 2 min at each light level. The oxygen evolution rate was then normalized to OD_730_.

Chlorophyll fluorescence was measured by a lightweight, hand-held fluorometer (AP-C 100; AquaPen, Drasov, Czech Republic). Cells at log phase with an OD_730_ of 0.6 to 0.7 were collected and then adapted to darkness for half an hour. Fv/Fm and rETR were all automatically detected by AP-C 100.

### RNA extraction and qRT-PCR analysis.

Cells at log phase with an OD_73_ of 0.6 to 0.7 were harvested by centrifugation at 10,000 × *g*, 4°C, for 10 min. Total RNA was isolated by using the TaKaRa MiniBEST universal RNA extraction kit, and the concentration of RNA was verified by Qubit 4 (Thermo Fisher). The cDNA was acquired by using the Prime Script RT reagent kit with genomic DNA (gDNA) eraser (TaKaRa, Beijing, China). The SYBR Premix *Ex Taq* II kit (TaKaRa) was used for qRT-PCR, and the reactions were run in a Light Cycler 480 II sequence detection system (Roche, Shanghai, China). Primers for target genes are all shown in Table S1, and *rnpA* (SYNPCC7002_A0989) was used as the reference gene (67). The results were analyzed according to the threshold cycle (2^−ΔΔCT^) method (68).

### Bioinformatics analysis.

One hundred thirty cyanobacterial genomes were downloaded from the NCBI database (update to 17 June 2019). The query sequences of SQR and PDO were based on our previous work (21). The SQR and PDO candidates were obtained by searching the database with the standalone BLASTP algorithm, using conventional criteria (E value of ≤1e^−5^, coverage of ≥45%, and identity of ≥30%). The candidates were analyzed by using ClustalW for alignment and MEGA 7.0 for neighbor-joining tree building with the following parameters: pairwise deletion, *p*-distance distribution, and bootstrap analysis of 1,000 repeats (69). The candidates in the same clade as the seeds were selected. The published flavocytochrome *c* sulfide dehydrogenase (FCSD) sequences were used as the outgroup of SQR, and the GloB (GloB hydroxyacylglutathione hydrolase) sequences were used as the outgroup of PDO.

## References

[1] Schmidtko S, Stramma L, Visbeck M 2017 Decline in global oceanic oxygen content during the past five decades. Nature 542:335–339. doi:10.1038/nature21399. 28202958

[2] Breitburg D, Levin LA, Oschlies A, Gregoire M, Chavez FP, Conley DJ, Garcon V, Gilbert D, Gutierrez D, Isensee K, Jacinto GS, Limburg KE, Montes I, Naqvi SWA, Pitcher GC, Rabalais NN, Roman MR, Rose KA, Seibel BA, Telszewski M, Yasuhara M, Zhang J 2018 Declining oxygen in the global ocean and coastal waters. Science 359:eaam7240. doi:10.1126/science.aam7240. 29301986

[3] Shepherd JG, Brewer PG, Oschlies A, Watson AJ 2017 Ocean ventilation and deoxygenation in a warming world: introduction and overview. Philos Trans R Soc A 375:20170240. doi:10.1098/rsta.2017.0240. PMC5559423 28784707

[4] Stramma L, Prince ED, Schmidtko S, Luo J, Hoolihan JP, Visbeck M, Wallace DWR, Brandt P, Körtzinger A 2012 Expansion of oxygen minimum zones may reduce available habitat for tropical pelagic fishes. Nat Clim Chang 2:33–37. doi:10.1038/nclimate1304.

[5] Keeling RE, Körtzinger A, Gruber N 2010 Ocean deoxygenation in a warming world. Annu Rev Mar Sci 2:199–229. doi:10.1146/annurev.marine.010908.163855. 21141663

[6] Diaz RJ, Rosenberg R 2008 Spreading dead zones and consequences for marine ecosystems. Science 321:926–929. doi:10.1126/science.1156401. 18703733

[7] Grantham BA, Chan F, Nielsen KJ, Fox DS, Barth JA, Huyer A, Lubchenco J, Menge BA 2004 Upwelling-driven nearshore hypoxia signals ecosystem and oceanographic changes in the northeast Pacific. Nature 429:749–754. doi:10.1038/nature02605. 15201908

[8] McCormick LR, Levin LA 2017 Physiological and ecological implications of ocean deoxygenation for vision in marine organisms. Philos Trans R Soc A 375:20160322. doi:10.1098/rsta.2016.0322. PMC5559417 28784712

[9] Gobler CJ, Baumann H 2016 Hypoxia and acidification in ocean ecosystems: coupled dynamics and effects on marine life. Biol Lett 12:20150976. doi:10.1098/rsbl.2015.0976. 27146441 PMC4892234

[10] Schunck H, Lavik G, Desai DK, Großkopf T, Kalvelage T, Löscher CR, Paulmier A, Contreras S, Siegel H, Holtappels M, Rosenstiel P, Schilhabel MB, Graco M, Schmitz RA, Kuypers MMM, Laroche J 2013 Giant hydrogen sulfide plume in the oxygen minimum zone off Peru supports chemolithoautotrophy. PLoS One 8:e68661. doi:10.1371/journal.pone.0068661. 23990875 PMC3749208

[11] Joyner-Matos J, Predmore BL, Stein JR, Leeuwenburgh C, Julian D 2010 Hydrogen sulfide induces oxidative damage to RNA and DNA in a sulfide-tolerant marine invertebrate. Physiol Biochem Zool 83:356–365. doi:10.1086/597529. 19327040 PMC4542154

[12] Cuevasanta E, Moller MN, Alvarez B 2017 Biological chemistry of hydrogen sulfide and persulfides. Arch Biochem Biophys 617:9–25. doi:10.1016/j.abb.2016.09.018. 27697462

[13] Bouillaud F, Blachier F 2011 Mitochondria and sulfide: a very old story of poisoning, feeding, and signaling? Antioxid Redox Signal 15:379–391. doi:10.1089/ars.2010.3678. 21028947

[14] Malone Rubright SL, Pearce LL, Peterson J 2017 Environmental toxicology of hydrogen sulfide. Nitric Oxide 71:1–13. doi:10.1016/j.niox.2017.09.011. 29017846 PMC5777517

[15] Grieshaber MK, Volkel S 1998 Animal adaptations for tolerance and exploitation of poisonous sulfide. Annu Rev Physiol 60:33–53. doi:10.1146/annurev.physiol.60.1.33. 9558453

[16] Miller SR, Bebout BM 2004 Variation in sulfide tolerance of photosystem II in phylogenetically diverse cyanobacteria from sulfidic habitats. Appl Environ Microbiol 70:736–744. doi:10.1128/aem.70.2.736-744.2004. 14766549 PMC348820

[17] Oren A, Padan E, Malkin S 1979 Sulfide inhibition of photosystem II in cyanobacteria (blue-green algae) and tobacco chloroplasts. Biochim Biophys Acta 546:270–279. doi:10.1016/0005-2728(79)90045-8. 109120

[18] Cohen Y, Jørgensen BB, Revsbech NP, Poplawski R 1986 Adaptation to hydrogen sulfide of oxygenic and anoxygenic photosynthesis among cyanobacteria. Appl Environ Microbiol 51:398–407. doi:10.1128/AEM.51.2.398-407.1986. 16346996 PMC238881

[19] Klatt JM, Haas S, Yilmaz P, de Beer D, Polerecky L 2015 Hydrogen sulfide can inhibit and enhance oxygenic photosynthesis in a cyanobacterium from sulfidic springs. Environ Microbiol 17:3301–3313. doi:10.1111/1462-2920.12791. 25630511

[20] Canfield DE, Stewart FJ, Thamdrup B, De Brabandere L, Dalsgaard T, Delong EF, Revsbech NP, Ulloa O 2010 A cryptic sulfur cycle in oxygen-minimum-zone waters off the Chilean coast. Science 330:1375–1378. doi:10.1126/science.1196889. 21071631

[21] Xia Y, Lü C, Hou N, Xin Y, Liu J, Liu H, Xun L 2017 Sulfide production and oxidation by heterotrophic bacteria under aerobic conditions. ISME J 11:2754–2766. doi:10.1038/ismej.2017.125. 28777380 PMC5702731

[22] Mehta-Kolte MG, Loutey D, Wang O, Youngblut MD, Hubbard CG, Wetmore KM, Conrad ME, Coates JD 2017 Mechanism of H_2_S oxidation by the dissimilatory perchlorate-reducing microorganism Azospira suillum PS. mBio 8:e02023-16. doi:10.1128/mBio.02023-16. 28223460 PMC5358917

[23] Luna-Sánchez M, Hidalgo-Gutiérrez A, Hildebrandt TM, Chaves-Serrano J, Barriocanal-Casado E, Santos-Fandila Á, Romero M, Sayed RK, Duarte J, Prokisch H, Schuelke M, Distelmaier F, Escames G, Acuña-Castroviejo D, López LC 2017 CoQ deficiency causes disruption of mitochondrial sulfide oxidation, a new pathomechanism associated with this syndrome. EMBO Mol Med 9:78–95. doi:10.15252/emmm.201606345. 27856619 PMC5210161

[24] Liu H, Xin Y, Xun L 2014 Distribution, diversity, and activities of sulfur dioxygenases in heterotrophic bacteria. Appl Environ Microbiol 80:1799–1806. doi:10.1128/AEM.03281-13. 24389926 PMC3957605

[25] Xin Y, Liu H, Cui F, Liu H, Xun L 2016 Recombinant Escherichia coli with sulfide:quinone oxidoreductase and persulfide dioxygenase rapidly oxidises sulfide to sulfite and thiosulfate via a new pathway. Environ Microbiol 18:5123–5136. doi:10.1111/1462-2920.13511. 27573649

[26] Cherney MM, Zhang Y, Solomonson M, Weiner JH, James MN 2010 Crystal structure of sulfide:quinone oxidoreductase from Acidithiobacillus ferrooxidans: insights into sulfidotrophic respiration and detoxification. J Mol Biol 398:292–305. doi:10.1016/j.jmb.2010.03.018. 20303979

[27] Grim SL, Dick GJ 2016 Photosynthetic versatility in the genome of Geitlerinema sp. PCC 9228 (formerly Oscillatoria limnetica ‘Solar Lake’), a model anoxygenic photosynthetic cyanobacterium. Front Microbiol 7:1546. doi:10.3389/fmicb.2016.01546. 27790189 PMC5061849

[28] Theissen U, Hoffmeister M, Grieshaber M, Martin W 2003 Single eubacterial origin of eukaryotic sulfide:quinone oxidoreductase, a mitochondrial enzyme conserved from the early evolution of eukaryotes during anoxic and sulfidic times. Mol Biol Evol 20:1564–1574. doi:10.1093/molbev/msg174. 12832624

[29] Dahl C, Franz B, Hensen D, Kesselheim A, Zigann R 2013 Sulfite oxidation in the purple sulfur bacterium Allochromatium vinosum: identification of SoeABC as a major player and relevance of SoxYZ in the process. Microbiology 159:2626–2638. doi:10.1099/mic.0.071019-0. 24030319

[30] Thorup C, Schramm A, Findlay AJ, Finster KW, Schreiber L 2017 Disguised as a sulfate reducer: growth of the Deltaproteobacterium Desulfurivibrio alkaliphilus by sulfide oxidation with nitrate. mBio 8:e00671-17. doi:10.1128/mBio.00671-17. 28720728 PMC5516251

[31] Klatt JM, de Beer D, Hausler S, Polerecky L 2016 Cyanobacteria in sulfidic spring microbial mats can perform oxygenic and anoxygenic photosynthesis simultaneously during an entire diurnal period. Front Microbiol 7:1973. doi:10.3389/fmicb.2016.01973. 28018309 PMC5156726

[32] Weissgerber T, Sylvester M, Kroninger L, Dahl C 2014 A comparative quantitative proteomic study identifies new proteins relevant for sulfur oxidation in the purple sulfur bacterium Allochromatium vinosum. Appl Environ Microbiol 80:2279–2792. doi:10.1128/AEM.04182-13. 24487535 PMC3993150

[33] Marcia M, Ermler U, Peng G, Michel H 2010 A new structure-based classification of sulfide:quinone oxidoreductases. Proteins 78:1073–1083. doi:10.1002/prot.22665. 20077566

[34] Shen J, Peng H, Zhang Y, Trinidad JC, Giedroc DP 2016 Staphylococcus aureus *sqr* encodes a type II sulfide:quinone oxidoreductase and impacts reactive sulfur speciation in cells. Biochemistry 55:6524–6534. doi:10.1021/acs.biochem.6b00714. 27806570 PMC5423654

[35] Li K, Xin Y, Xuan G, Zhao R, Liu H, Xia Y, Xun L 2019 Escherichia coli uses separate enzymes to produce H_2_S and reactive sulfane sulfur from L-cysteine. Front Microbiol 10:298. doi:10.3389/fmicb.2019.00298. 30873134 PMC6401616

[36] Greiner R, Palinkas Z, Basell K, Becher D, Antelmann H, Nagy P, Dick TP 2013 Polysulfides link H_2_S to protein thiol oxidation. Antioxid Redox Signal 19:1749–1765. doi:10.1089/ars.2012.5041. 23646934 PMC3837443

[37] Iciek M, Kowalczyk-Pachel D, Bilska-Wilkosz A, Kwiecien I, Gorny M, Wlodek L 2015 S-sulfhydration as a cellular redox regulation. Biosci Rep 36:e00304. doi:10.1042/BSR20150147. 26607972 PMC5293568

[38] Paul BD, Snyder SH 2015 Protein sulfhydration. Methods Enzymol 555:79–90. doi:10.1016/bs.mie.2014.11.021. 25747476

[39] Huang S, Wilhelm SW, Harvey HR, Taylor K, Jiao N, Chen F 2012 Novel lineages of Prochlorococcus and Synechococcus in the global oceans. ISME J 6:285–297. doi:10.1038/ismej.2011.106. 21955990 PMC3260499

[40] Shih PM 2019 Early cyanobacteria and the innovation of microbial sunscreens. mBio 10:e01262-19. doi:10.1128/mBio.01262-19. 31186332 PMC6561034

[41] Cohen Y, Jørgensen BB, Padan E, Shilo M 1975 Sulphide-dependent anoxygenic photosynthesis in the cyanobacterium Oscillatoria limnetica. Nature 257:489–492. doi:10.1038/257489a0. PMC235807 808537

[42] Klatt JM, Al-Najjar MA, Yilmaz P, Lavik G, de Beer D, Polerecky L 2015 Anoxygenic photosynthesis controls oxygenic photosynthesis in a cyanobacterium from a sulfidic spring. Appl Environ Microbiol 81:2025–2031. doi:10.1128/AEM.03579-14. 25576611 PMC4345360

[43] Zhao Z, Gonsior M, Schmitt-Kopplin P, Zhan Y, Zhang R, Jiao N, Chen F 2019 Microbial transformation of virus-induced dissolved organic matter from picocyanobacteria: coupling of bacterial diversity and DOM chemodiversity. ISME J 13:2551–2565. doi:10.1038/s41396-019-0449-1. 31227815 PMC6776026

[44] Chen F, Wang K, Huang S, Cai H, Zhao M, Jiao N, Wommack KE 2009 Diverse and dynamic populations of cyanobacterial podoviruses in the Chesapeake Bay unveiled through DNA polymerase gene sequences. Environ Microbiol 11:2884–2892. doi:10.1111/j.1462-2920.2009.02033.x. 19703219

[45] Wang Y, Liu Y, Wang J, Luo T, Zhang R, Sun J, Zheng Q, Jiao N 2019 Seasonal dynamics of bacterial communities in the surface seawater around subtropical Xiamen Island, China, as determined by 16S rRNA gene profiling. Mar Pollut Bull 142:135–144. doi:10.1016/j.marpolbul.2019.03.035. 31232286

[46] Li Y, Tang K, Zhang L, Zhao Z, Xie X, Chen CA, Wang D, Jiao N, Zhang Y 2018 Coupled carbon, sulfur, and nitrogen cycles mediated by microorganisms in the water column of a shallow-water hydrothermal ecosystem. Front Microbiol 9:2718. doi:10.3389/fmicb.2018.02718. 30555427 PMC6282030

[47] Koch T, Dahl C 2018 A novel bacterial sulfur oxidation pathway provides a new link between the cycles of organic and inorganic sulfur compounds. ISME J 12:2479–2491. doi:10.1038/s41396-018-0209-7. 29930335 PMC6155103

[48] Shah V, Zhao X, Lundeen RA, Ingalls AE, Nicastro D, Morris RM 2019 Morphological plasticity in a sulfur-oxidizing marine bacterium from the SUP05 clade enhances dark carbon fixation. mBio 10:e00216-19. doi:10.1128/mBio.00216-19. 31064824 PMC6509183

[49] Bibli SI, Luck B, Zukunft S, Wittig J, Chen W, Xian M, Papapetropoulos A, Hu J, Fleming I 2018 A selective and sensitive method for quantification of endogenous polysulfide production in biological samples. Redox Biol 18:295–304. doi:10.1016/j.redox.2018.07.016. 30077923 PMC6083819

[50] Lloyd D 2006 Hydrogen sulfide: clandestine microbial messenger? Trends Microbiol 14:456–462. doi:10.1016/j.tim.2006.08.003. 16908154

[51] Li H, Li J, Lu C, Xia Y, Xin Y, Liu H, Xun L, Liu H 2017 FisR activates sigma(54)-dependent transcription of sulfide-oxidizing genes in Cupriavidus pinatubonensis JMP134. Mol Microbiol 105:373–384. doi:10.1111/mmi.13725. 28612361

[52] Shimizu T, Shen J, Fang M, Zhang Y, Hori K, Trinidad JC, Bauer CE, Giedroc DP, Masuda S 2017 Sulfide-responsive transcriptional repressor SqrR functions as a master regulator of sulfide-dependent photosynthesis. Proc Natl Acad Sci U S A 114:2355–2360. doi:10.1073/pnas.1614133114. 28196888 PMC5338557

[53] Luebke JL, Shen J, Bruce KE, Kehl-Fie TE, Peng H, Skaar EP, Giedroc DP 2014 The CsoR-like sulfurtransferase repressor (CstR) is a persulfide sensor in Staphylococcus aureus. Mol Microbiol 94:1343–1360. doi:10.1111/mmi.12835. 25318663 PMC4264537

[54] Hou N, Yan Z, Fan K, Li H, Zhao R, Xia Y, Xun L, Liu H 2019 OxyR senses sulfane sulfur and activates the genes for its removal in Escherichia coli. Redox Biol 26:101293. doi:10.1016/j.redox.2019.101293. 31421411 PMC6831875

[55] Chen Z, Zhang X, Li H, Liu H, Xia Y, Xun L 2018 The complete pathway for thiosulfate utilization in Saccharomyces cerevisiae. Appl Environ Microbiol 84:e01241-18. doi:10.1128/AEM.01241-18. 30217845 PMC6210100

[56] Olson KR, Straub KD 2016 The role of hydrogen sulfide in evolution and the evolution of hydrogen sulfide in metabolism and signaling. Physiology (Bethesda) 31:60–72. doi:10.1152/physiol.00024.2015. 26674552

[57] Olson KR 2018 H_2_S and polysulfide metabolism: conventional and unconventional pathways. Biochem Pharmacol 149:77–90. doi:10.1016/j.bcp.2017.12.010. 29248597

[58] Kimura H 2015 Signaling molecules: hydrogen sulfide and polysulfide. Antioxid Redox Signal 22:362–376. doi:10.1089/ars.2014.5869. 24800864 PMC4307032

[59] Olson KR 2019 Hydrogen sulfide, reactive sulfur species and coping with reactive oxygen species. Free Radic Biol Med 140:74–83. doi:10.1016/j.freeradbiomed.2019.01.020. 30703482

[60] Zhang CC, Zhou CZ, Burnap RL, Peng L 2018 Carbon/nitrogen metabolic balance: lessons from cyanobacteria. Trends Plant Sci 23:1116–1130. doi:10.1016/j.tplants.2018.09.008. 30292707

[61] Jiang YL, Wang XP, Sun H, Han SJ, Li WF, Cui N, Lin GM, Zhang JY, Cheng W, Cao DD, Zhang ZY, Zhang CC, Chen Y, Zhou CZ 2018 Coordinating carbon and nitrogen metabolic signaling through the cyanobacterial global repressor NdhR. Proc Natl Acad Sci U S A 115:403–408. doi:10.1073/pnas.1716062115. 29279392 PMC5777067

[62] Latifi A, Ruiz M, Zhang CC 2009 Oxidative stress in cyanobacteria. FEMS Microbiol Rev 33:258–278. doi:10.1111/j.1574-6976.2008.00134.x. 18834454

[63] Stevens SE, Porter RD 1980 Transformation in Agmenellum quadruplicatum. Proc Natl Acad Sci U S A 77:6052–6056. doi:10.1073/pnas.77.10.6052. 16592896 PMC350211

[64] Ruffing AM, Jensen TJ, Strickland LM 2016 Genetic tools for advancement of Synechococcus sp. PCC 7002 as a cyanobacterial chassis. Microb Cell Fact 15:190. doi:10.1186/s12934-016-0584-6. 27832791 PMC5105302

[65] Xia Y, Li K, Li J, Wang T, Gu L, Xun L 2019 T5 exonuclease-dependent assembly offers a low-cost method for efficient cloning and site-directed mutagenesis. Nucleic Acids Res 47:e15. doi:10.1093/nar/gky1169. 30462336 PMC6379645

[66] Lü C, Xia Y, Liu D, Zhao R, Gao R, Liu H, Xun L 2017 Cupriavidus necator H16 uses flavocytochrome c sulfide dehydrogenase to oxidize self-produced and added sulfide. Appl Environ Microbiol 83:e01610-17. doi:10.1128/AEM.01610-17. 28864655 PMC5666145

[67] Szekeres E, Sicora C, Dragoş N, Drugă B 2014 Selection of proper reference genes for the cyanobacterium Synechococcus PCC 7002 using real-time quantitative PCR. FEMS Microbiol Lett 359:102–109. doi:10.1111/1574-6968.12574. 25115691

[68] Livak KJ, Schmittgen TD 2001 Analysis of relative gene expression data using real-time quantitative PCR and the 2(-delta delta C(T)) method. Methods 25:402–408. doi:10.1006/meth.2001.1262. 11846609

[69] Kumar S, Stecher G, Tamura K 2016 MEGA7: Molecular Evolutionary Genetics Analysis version 7.0 for bigger datasets. Mol Biol Evol 33:1870–1874. doi:10.1093/molbev/msw054. 27004904 PMC8210823

