# Supplementary figures and images for: Synechococcus sp. Strain PCC7002 Uses Sulfide:Quinone Oxidoreductase To Detoxify Exogenous Sulfide and To Convert Endogenous Sulfide to Cellular Sulfane Sulfur
Source: mBio. 2020 Feb 25;11(1):e03420-19. doi: 10.1128/mBio.03420-19 (PMC7042703; doi:10.1128/mBio.03420-19)

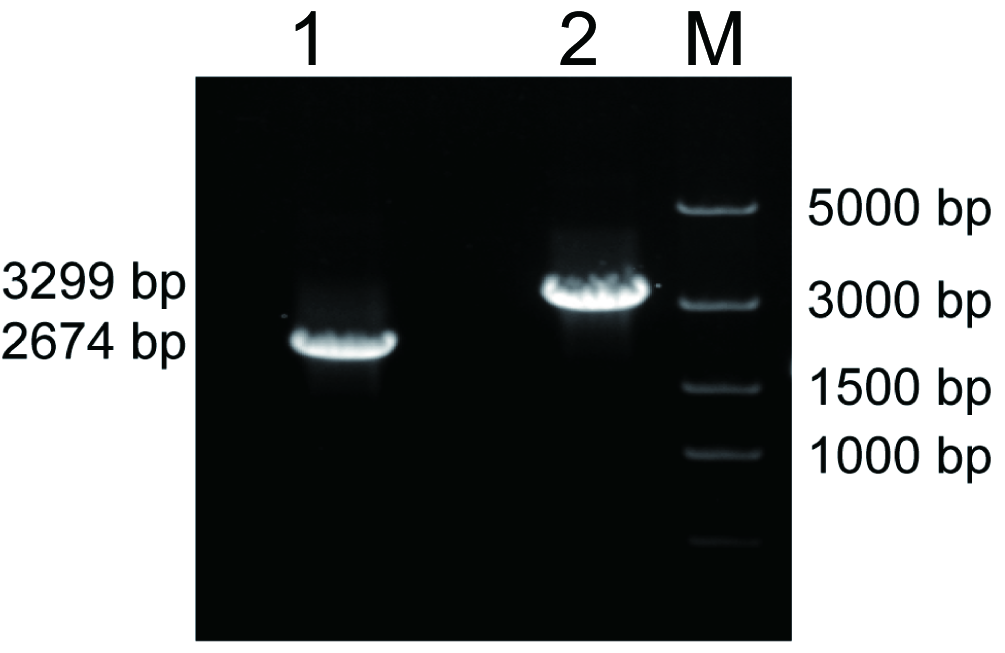

Supplement: FIG S1 [file mBio.03420-19-sf001.tif]

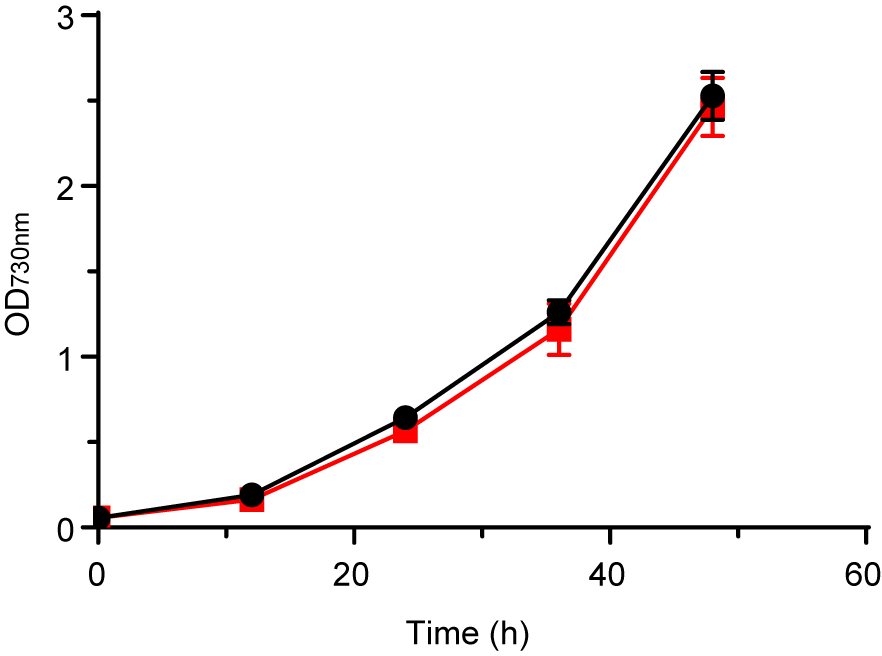

Supplement: FIG S2 [file mBio.03420-19-sf002.tif]

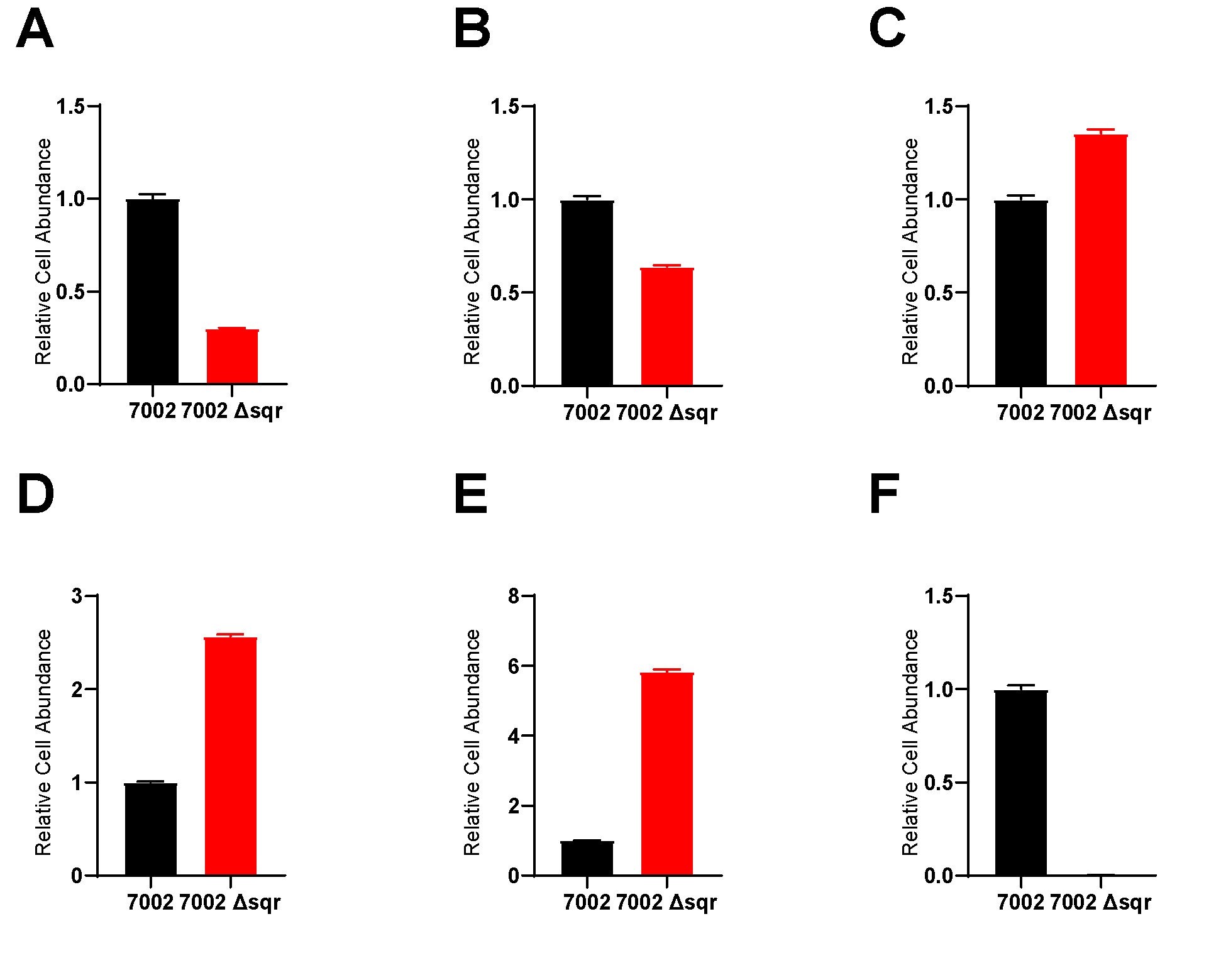

Supplement: FIG S3 [file mBio.03420-19-sf003.tif]

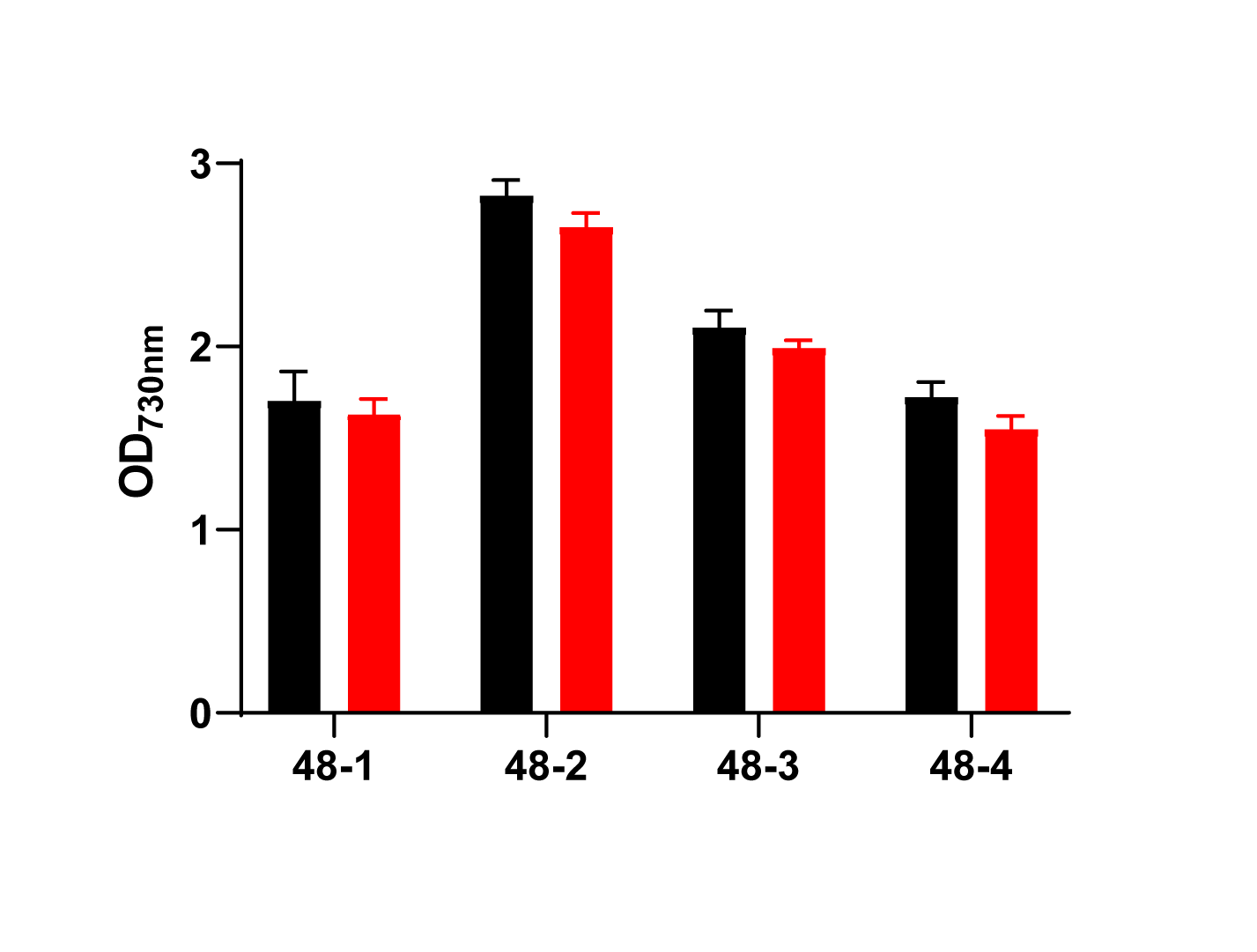

Supplement: FIG S4 [file mBio.03420-19-sf004.tif]

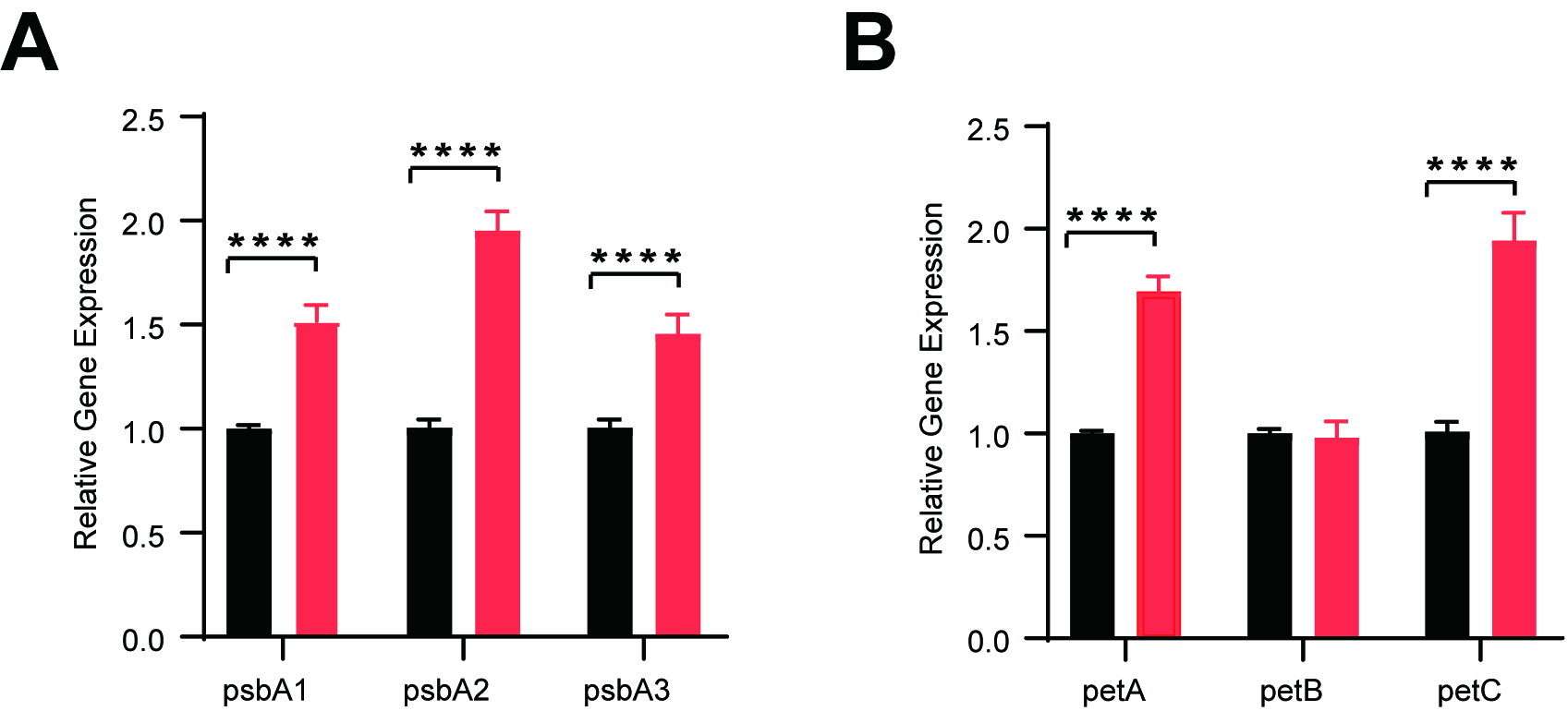

Supplement: FIG S5 [file mBio.03420-19-sf005.tif]

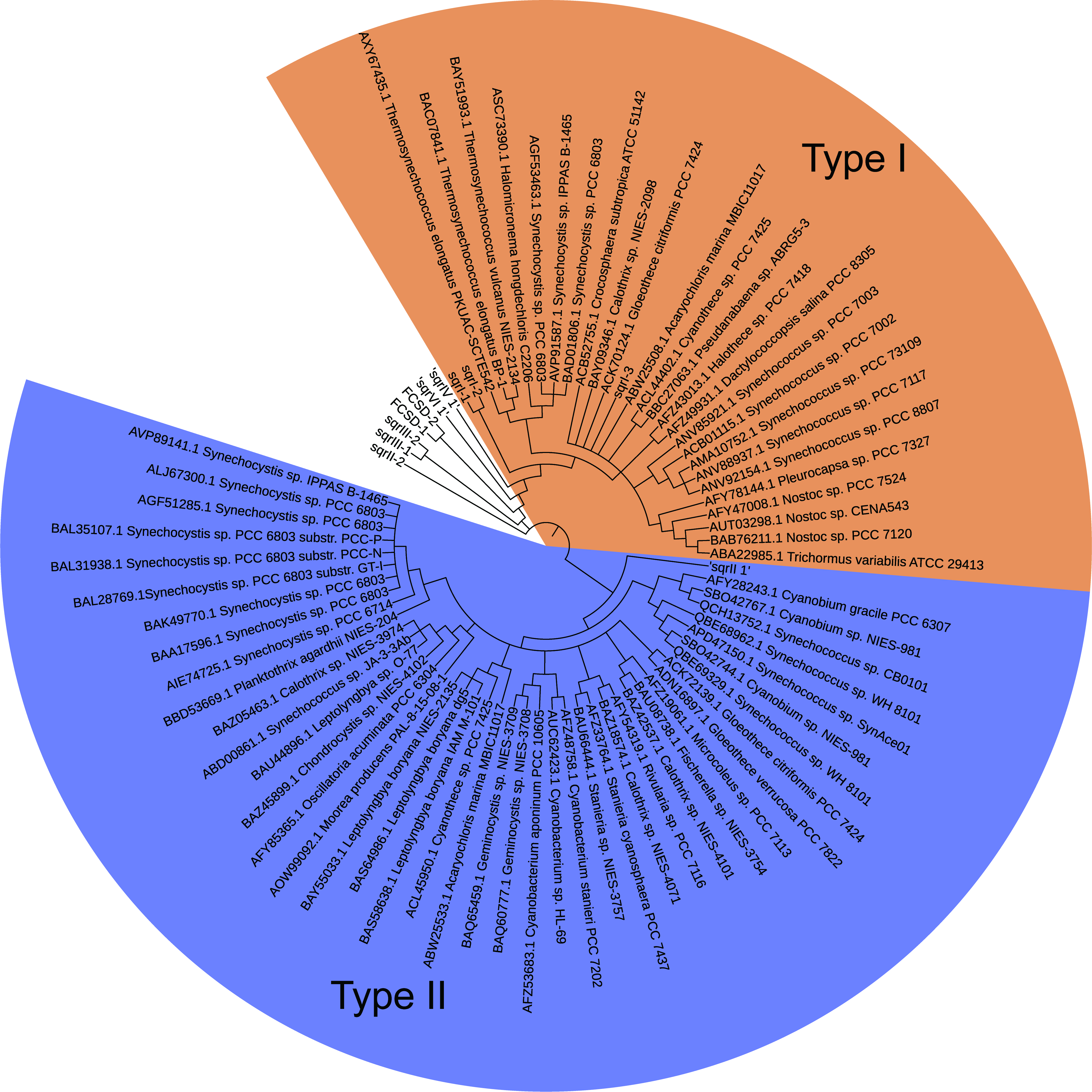

Supplement: FIG S6 [file mBio.03420-19-sf006.tif]
