# Supplementary material for: Synechococcus sp. Strain PCC7002 Uses Sulfide:Quinone Oxidoreductase To Detoxify Exogenous Sulfide and To Convert Endogenous Sulfide to Cellular Sulfane Sulfur
Source: mBio. 2020 Feb 25;11(1):e03420-19. doi: 10.1128/mBio.03420-19 (PMC7042703; doi:10.1128/mBio.03420-19)
Supplement: TABLE S1 [file mBio.03420-19-st001.docx]

**Table S1. Strains and plasmids used in this study**

| Strain or plasmid | Description/characteristic | Source/reference |
| --- | --- | --- |
| Strains |  |  |
| PCC7002 | Wilde type | This study |
| PCC7002△*sqr* | PCC7002 with *sqr* deletion | This study |
| PCC7002△*sqr::sqr* | PCC7002 **△***sqr* with *sqr* insertion into the neutral site 1 (NS1) on the chromosome  (NS1: SYNPCC7002_A0933) | This study |
| *E. coli* DH5α | Cloning strain | Novagen |
| *E. coli* BL21(DE3) | Cloning strain | Novagen |
| Plasmids |  |  |
| pBBR1MCS-5 | expression vector | (67) |
| pJET1.2-Blunt | Cloning vector | Thermo Fisher |
| pJET-*sqr*-del | *sqr* deletion vector | This study |
| pJET-*sqr*-com | *sqr* complementation vector | This study |
| pBBR5-*sqr* | pBBR1MCS-5 containing *sqr* from PCC7002 | This study |
| pBBR5-*sqr-pdo* | pBBR1MCS-5 containing *sqr* and *pdo* from PCC7002 | This study |
