# Supplementary material for: Synechococcus sp. Strain PCC7002 Uses Sulfide:Quinone Oxidoreductase To Detoxify Exogenous Sulfide and To Convert Endogenous Sulfide to Cellular Sulfane Sulfur
Source: mBio. 2020 Feb 25;11(1):e03420-19. doi: 10.1128/mBio.03420-19 (PMC7042703; doi:10.1128/mBio.03420-19)
Supplement: TABLE S2 [file mBio.03420-19-st002.docx]

**Table S2. Primers used in this study**

| **Primer name** | **Sequence** | **Description** |
| --- | --- | --- |
| *sqr*-*del*-1 | AGCACACATCCCAACAACAGC | *sqr* deletion |
| *sqr*-*del*-2 | CCAACTTAATCGCCTTGCAGCAGAGGGTAACAGTGTGGTCGGT |  |
| *sqr*-*del*-3 | ACCGACCACACTGTTACCCTCTGCTGCAAGGCGATTAAGTTGG |  |
| *sqr*-*del*-4 | GTGGGGATTGGTGTGGCTTCATACGACAGGTTTCCCGACTGG |  |
| *sqr*-*del*-5 | CCAGTCGGGAAACCTGTCGTATGAAGCCACACCAATCCCCAC |  |
| *sqr*-*del*-6 | CATTTGTGTAGCGTGAATCAGG |  |
| *sqr*-com-1 | TCATGTGGTGGAATTGCCAG | *sqr* complementation |
| *sqr*-com-2 | TTCCGACTTGTGGTGAAGCAGTACCGAGTGCGATCAACGGACACAAACCAT |  |
| *sqr*-com-3 | ATGGTTTGTGTCCGTTGATCGCACTCGGTACTGCTTCACCACAAGTCGGAA |  |
| *sqr*-com-4 | AGTGTCCCCTTATACACAAGGATGTGTAGCGTGAATCAGGATTGG |  |
| *sqr*-com-5 | CCAATCCTGATTCACGCTACACATCCTTGTGTATAAGGGGACACT |  |
| *sqr*-com-6 | CTGAGGCAGATTGCGGCTTACTCCGGGAAGCCGATCTCGGCT |  |
| *sqr*-com-7 | AGCCGAGATCGGCTTCCCGGAGTAAGCCGCAATCTGCCTCAG |  |
| *sqr*-com-8 | ACACCACCATTGATGGGGTTCATCG |  |
| pbr-*sqr*-F | CACACAGGAAACAGCTATGGCTCATATTGTTGTAATCGGTGC | *sqr* expression |
| pbr-*sqr*-R | TTCCATTCGCCATTCATTAGTCTGGAGGAAGGGGAC |  |
| pbr-*sqr-pdo*-1 | TTAGTCTGGAGGAAGGGGAC | *sqr-pdo* expression |
| pbr-*sqr-pdo*-2 | GTCCCCTTCCTCCAGACTAATGAACCATGCTCTTTCGCCAAC |  |
| pbr-*sqr-pdo*-3 | TTCCATTCGCCATTCAGAGTTAAATATTGCCACAGGCT |  |
| *PsbA1*-RT-F | TCATGATCGTATTCCAGGCA | RT-qPCR primers |
| *PsbA1*-RT-R | TACGAGAGAACCGTGCATTG |  |
| *PsbA2*-RT-F | AAACCGTTCAGGTTGAATGC |  |
| *PsbA2*-RT-R | CGGTCGTTTGATCTTCCAAT |  |
| *PsbA3*-RT-F | GCTTGGCCAGTGATTGGTAT |  |
| *PsbA3*-RT-R | CCGTCCTTGGGAATCTAACA |  |
| *RbcS*-RT-F | GTCGAAACCAACAACACGGA |  |
| *RbcS*-RT-R | ACTCCTGAACTCCACCACTG |  |
| *RbcL*-RT-F | CAAACTGGAGGCAGGAATCG |  |
| *RbcL*-RT-R | AGATCGTTCTCGCGGTGTAT |  |
| *RbcX*-RT-F | CGTCAGGATTCTGAGGACGA |  |
| *RbcX*-RT-R | GTCAGCAGCTTAGTGAAACCA |  |
| *PetA*-RT-F | GATCGTCTGTGCCAACTGTC |  |
| *PetA*-RT-R | GAACCTGCTGTTGGCTATGG |  |
| *PetB*-RT-F | TGCTACGGTGGGTTTGTAGT |  |
| *PetB*-RT-R | ATCCAAGCGATCTCTGACGA |  |
| *PetC*-RT-F | TGGTGGCGTGATTGCTAAAG |  |
| *PetC*-RT-R | ACCAAGGTGGGTACAGATGG |  |
| *Tkt*-RT-F | CTTAGCCGCTTCAATGGCTT |  |
| *Tkt*-RT-R | GCCCTCTACGACGATAACCA |  |
| *Rnp*A-RT-F | GCCCCAACCAAAATTGGCATCAG |  |
| *Rnp*A-RT-R | TACCGCACAGCAATGACGATGTG |  |
| com-wild-F | AGCAGGTACCGATCACATTG |  |
| com-wild-R | CTGTAGGCAAAGGGTAGCTG |  |
| com-mut-F | TGATGGTCGGAAGAGGCATA |  |
| com-mut-R | TAATGTCGGGCAATCAGGTG |  |
